# How Safe Are COVID-19 Vaccines in Individuals with Immune-Mediated Inflammatory Diseases? The SUCCEED Study

**DOI:** 10.3390/vaccines12091027

**Published:** 2024-09-08

**Authors:** Olga Tsyruk, Gilaad G. Kaplan, Paul R. Fortin, Carol A Hitchon, Vinod Chandran, Maggie J. Larché, Antonio Avina-Zubieta, Gilles Boire, Ines Colmegna, Diane Lacaille, Nadine Lalonde, Laurie Proulx, Dawn P. Richards, Natalie Boivin, Christopher DeBow, Lucy Kovalova-Wood, Deborah Paleczny, Linda Wilhelm, Luck Lukusa, Daniel Pereira, Jennifer LF. Lee, Sasha Bernatsky, on behalf of the SUCCEED Investigative Team

**Affiliations:** 1 Department of Medicine, McGill University, Montreal, QC H4A 3J1, Canada; 2 Division of Gastroenterology and Hepatology, Departments of Medicine and Community Health Sciences, University of Calgary, Calgary, AB T2N 4Z6, Canada; 3 Centre de Recherche ARThrite-UL, Division of Rheumatology, Department of Medicine, CHU de Québec-Université Laval, Québec City, QC G1V 4G2, Canada; 4 Axe Maladies Infectieuses et Immunitaires, Centre de Recherche du CHU de Québec-Université Laval, Québec City, QC G1V 4G2, Canada; 5 Department of Internal Medicine, Max Rady College of Medicine, Rady Faculty of Health Sciences, University of Manitoba, Winnipeg, MB R3A 1R9, Canada; 6 Schroeder Arthritis Institute, Krembil Research Institute, University Health Network, Toronto, ON M5T 2S8, Canada; 7 Division of Rheumatology, Department of Medicine, University of Toronto, Toronto, ON M5S 3H2, Canada; 8 Division of Rheumatology, Department of Medicine, McMaster University, Hamilton, ON L8N 3Z5, Canada; 9 Arthritis Research Canada and Division of Rheumatology, University of British Columbia, Vancouver, BC V5Y 3P2, Canada; 10 Division of Rheumatology, Department of Medicine, Université de Sherbrooke, Sherbrooke, QC J1H 5N4, Canada; 11 The Research Institute of the McGill University Health Centre, McGill University, Montreal, QC H4A 3J1, Canada; 12 Canadian Arthritis Patient Alliance, Toronto, ON L6A 4Z6, Canada

**Keywords:** COVID-19 vaccine safety, immune-mediated inflammatory diseases, serious adverse events

## Abstract

We were tasked by Canada’s COVID-19 Immunity Task Force to describe severe adverse events (SAEs) associated with emergency department (ED) visits and/or hospitalizations in individuals with immune-mediated inflammatory diseases (IMIDs). At eight Canadian centres, data were collected from adults with rheumatoid arthritis (RA), axial spondyloarthritis (AxS), systemic lupus (SLE), psoriatic arthritis (PsA), and inflammatory bowel disease (IBD). We administered questionnaires, analyzing SAEs experienced within 31 days following SARS-CoV-2 vaccination. About two-thirds (63%) of 1556 participants were female; the mean age was 52.5 years. The BNT162b2 (Pfizer) vaccine was the most common, with mRNA-1273 (Moderna) being second. A total of 49% of participants had IBD, 27.4% had RA, 14.3% had PsA, 5.3% had SpA, and 4% had SLE. Twelve (0.77% of 1556 participants) SAEs leading to an ED visit or hospitalization were self-reported, occurring in 11 participants. SAEs included six (0.39% of 1556 participants) ED visits (including one due to Bell’s Palsy 31 days after first vaccination) and six (0.39% of 1556 participants) hospitalizations (including one due to Guillain-Barré syndrome 15 days after the first vaccination). Two SAEs included pericarditis, one involved SLE (considered a serious disease flare), and one involved RA. Thus, in the 31 days after SARS-CoV-2 vaccination in our IMID sample, very few serious adverse events occurred. As SARS-CoV2 continues to be a common cause of death, our findings may help optimize vaccination acceptance.

## 1. Introduction

Severe acute respiratory syndrome coronavirus 2 (SARS-CoV-2) is now endemic and remains a global concern. Vaccines are effective in reducing severe SARS-CoV-2 infection and deaths in the general population [1,2,3]. However, the original vaccine safety trials largely excluded immunocompromised individuals who have increased risk of a serious SARS-CoV-2 infection [1,2].

Immune-mediated inflammatory diseases (IMIDs), such as rheumatoid arthritis (RA), inflammatory bowel disease (IBD), axial spondyloarthritis (AxS), psoriasis/psoriatic arthritis (PsO⁄PsA), systemic lupus erythematosus (SLE), and other conditions, affect up to 10% of North Americans [4,5,6]. Many individuals with an IMID take immune-suppressing medications, placing them at a higher risk for COVID-19-related death, critical care need, and hospital admissions [7]. Information on safety helps in decision making regarding vaccination against SARS-CoV-2 in individuals with an IMID [8]. SARS-CoV2 continues to be a common cause of death in the general population, and providing robust, detailed data on severe adverse events may help optimize vaccination acceptance by people with IMIDs.

The surveillance of responses to COVID-19 vaccines in systemic immune-mediated inflammatory diseases (SUCCEED) project was launched in 2021 to capture information on vaccine safety and effectiveness as a part of Canada’s COVID Immunity Task Force initiative (CITF, created by the government of Canada in 2020) to inform the National Advisory Committee on Immunization (NACI) and the Public Health Agency of Canada (PHAC) regarding Canada’s vaccine strategies for individuals with an IMID. One of the primary objectives of this study was to describe vaccine safety, particularly severe adverse events (SAEs), defined as events associated with emergency department (ED) visits and/or hospitalizations.

## 2. Methods

Consenting adults with RA, IBD, AxS, PsO/PsA, and SLE were recruited from clinical centres in Vancouver, Calgary, Winnipeg, Montreal, Quebec City, Sherbrooke, Toronto, and Hamilton. Initial recruitment (in all centres except Vancouver, Montreal, and Sherbrooke) began in early 2021, a few months after Canada began vaccinating against SARS-CoV-2 (mostly with mRNA formulations), and ended in March 2023. Patients were recruited by research staff during in-person patient visits at each clinical investigator’s centre. No selection criteria were used to approach patients other than the study’s inclusion criteria; patients were approached consecutively as they presented for clinical care. We were not allowed by our ethics committee to collect information about those who refused. Treating physicians could also obtain permission from patients (during a telehealth or in-person visit) for the research coordinator to contact them via email or telephone to explain the study and obtain consent from patients.

Recruitment at most centres targeted the assessment timepoints of pre-doses 1 and 2 (for mRNA formulations, which were anticipated to be the vast majority) at 2–4 weeks and then 3 and 6 months later. CITF funding was rolled out in April 2021, and as it became clear that patients would be receiving 3rd and subsequent boosters, we amended our protocol to include those time points. We anticipated that the vast majority (about 90% of our target of approximately 2000) of subjects would be on a disease-modifying agent and/or biologic and/or systemic corticosteroid, though this was not a criterion, and we anticipated that about 10% of our participants with an IMID might be on minimal (e.g., hydroxychloroquine or topical agents) or no IMID therapy. Exclusion criteria were individuals who had not been vaccinated for COVID-19 for a duration longer than 6 months prior to recruitment and/or unvaccinated individuals (unless they planned to be vaccinated shortly).

Participants provided baseline and follow-up questionnaires (paper or electronic) on past COVID-19 infections (including dates and treatments), COVID-19 vaccinations (including dates and type), and clinical history (type of IMID, date of diagnosis, and medications). Post-enrolment, participants were asked to contact the research team if they received additional vaccine doses, developed a COVID-19 infection, or had a vaccine-related adverse event. Participants were also contacted at 3, 6, and 12 months to provide updated information on medications and confirm whether or not they had an additional COVID-19 infection, vaccinations, or adverse event up to the end of the study. The questionnaire included a separate form where participants self-reported any adverse events that they believed were related to the vaccine. To ensure completeness and accuracy in reporting, we reviewed all self-reported events and requested additional information or verification from the participating center if any event required clarification, including confirmation through the patient’s medical records. In addition, to ensure no events were missed, we also included a section in the questionnaire to collect data on ED visits/hospitalizations for any reason throughout the duration of the study, regardless of whether they were related to the vaccine.

Recorded baseline information included demographic characteristics (age, sex, and race/ethnicity), clinical factors (type of IMID, disease duration, and drugs) and COVID-19 vaccination (dates and types of vaccine). Follow-up questionnaires at 3- to 6-month intervals were completed by participants to update their clinical history (i.e., disease activity, drugs, COVID-19 testing), health care use (physician visits, emergency room visits, and hospitalizations, including reasons for each), and COVID-19 vaccination history (type and date).

The number of ED visits and hospitalizations occurring within 31 days of vaccine administration were described (separately) following each vaccination dose, focusing specifically on severe adverse events. Adverse events post-vaccination are typically monitored and reported within 28–31 days in many vaccination studies [1,2]. The 31-day timeframe was chosen to include the events happening within a month post-vaccination as events within this period have a higher likelihood of being attributable to the effect of the vaccine and being correctly remembered by participants. We collected AEs based on Health Canada’s official Adverse Events Following Immunization (AEFI) form. In this form, severe adverse events are defined as vaccine-related AEs that either led to an ED visit or a hospitalization [9]. Among all events, it was determined how many were due to a severe IMID flare and how many were events may have represented a non-flare severe vaccine side effect (e.g., Bell’s Palsy, cardiac events, etc.). This allowed us to report (a) the number of severe disease flares requiring an ED visit or hospitalization within 31 days post-vaccination and (b) the number of ED visits or hospitalizations due to severe vaccine adverse events.

To describe patient demographics, age, and disease duration at study entry, we used means and standard deviations. Other characteristics were described in terms of frequency (N, percent).

A virtual meeting was held with people with lived experiences of IMIDs, where results were presented to them. Their feedback was collected regarding key takeaways and whether the results align with their experiences. These comments were incorporated into the paper discussion. People with lived experiences had also been involved in the initial grant writing process, planning process, the process of conducting the study, and in the interpretation of preliminary results at every step of the study.

## 3. Results

The demographic and clinical characteristics of the study sample are presented in Table 1. About two-thirds (63%) of 1556 participants were female; the mean age was 52.5 years. The BNT162b2 vaccine accounted for 75% of first/second doses, 67.8% of third doses, 63.3% of fourth doses, and 62% of fifth doses. mRNA-1273 was the second most common vaccine. Forty-nine percent of participants had IBD, 27.4% had RA, 14.3% had PsO/PsA, 5.3% had AxS, and 4% had SLE.

Presented in Table 2, there were 12 (0.77%; 95% CI: 0.34%, 1.2%) self-reported SAEs leading to an ED visit or hospitalization, which occurred in 11 participants; one participant reported 2 SAEs (pericarditis due to a potential SLE flare and labyrinthitis). There were six adverse events requiring ED visits. These included one case of Bell’s Palsy (31 days after the first vaccine dose), one serious allergic reaction of hives and a severe rash (within 24 h after the first vaccine dose), one episode of labyrinthitis (27 days after the third vaccine dose), one case of severe menstrual bleeding (4 days after the second vaccine dose) and one case of pericarditis (24 days after the third vaccine dose). This pericarditis event may have represented a case of a disease flare in a person with SLE given the concomitant new presence of other SLE symptoms. This participant with SLE also reported a second event, which was labyrinthitis; this occurred 3 days following the diagnosis of pericarditis. The remaining sixth event was a case of pericarditis which was not specifically labelled by the individual (who had RA) as vaccine-related and occurred 21 days after the second vaccine dose. With the exception of the above potential disease flare in the participant with SLE, no other disease flares requiring an ED visit/hospitalization within 31 days of vaccination were recorded.

There were six hospitalizations (unrelated to the above non-hospitalized ED visits) which occurred within 31 days following a COVID-19 vaccination, and all were labeled by the participants as a vaccine-related adverse event; these included one case of Guillain–Barre syndrome (15 days after the first vaccine dose), idiopathic thrombocytopenic purpura (within 31 days after the second vaccine dose), one episode of atrial fibrillation (in an elderly RA patient with a known prior heart disease 21 days after the third vaccine dose), one episode of transient multifactorial renal failure, possibly triggered by diarrhea (9 days after the third vaccine dose), one newly onset migraine with aura (one day after the fourth vaccine dose), and one diverticulosis flare in a patient who presented to the hospital multiple times for recurrent diverticulosis (1 day after the third vaccine dose). There were two other hospitalizations within 31 days of a COVID-19 vaccine which were not specifically labelled by the participants as vaccine-related; these included one case of shingles (27 days after the fourth vaccine dose) and one episode of epiploic appendagitis (torsion of an outpouching of peritoneal fat 24 days after the third vaccine dose).

Among the 12 self-reported vaccine-related SAEs, 7 were experienced by participants <65 years old. Three events occurred after the first vaccine dose, three after the second dose, five after the third dose, and one after the fourth dose. No deaths occurred within 31 days after vaccination. The details about each participant are presented in Table 3.

## 4. Discussion

In this large, longitudinal study, participants with IMIDs reported the frequency of SAEs post COVID-19 vaccination. In summary, the number of serious adverse events in our study was relatively low, with 12 SAEs being reported within 31 days by 11 individuals, which is reassuring to policymakers, clinicians, and, most importantly, people with IMIDs. Myocarditis, cardiogenic shock, myocardial infarction, or arterial/venous thrombosis have all been reported in the general population after vaccination with mRNA COVID-19 vaccines and other products as well [1,10,11]. Our participants did not identify any ED visits or hospitalizations within 31 days of vaccination that were related to these events. Our results are consistent with what has been reported in the literature on IMIDs [12,13,14,15,16,17]. A study investigating COVID-19 vaccine safety in RA and SLE samples found that 1.8% of their participants experienced an SAE [12]. A larger study by Naveen et al. showed that in their RA sample, the frequency of a major AE varied between 3.8 and 5.1% depending on disease activity [13]. Botwin et al. showed that in their IBD sample, the AE frequency was similar to what was reported in the general population, with only a few SAEs requiring hospitalization [14]. Pellegrino et al. did not find any SAEs in their IBD sample [15], similar to Cruz et al. in their RA sample [16]. It is important to note that most of the previously published literature on safety had small sample sizes of a few hundred participants, and what distinguishes our study is that our participant sample size was significantly larger, encompassing a diverse group of IMID types.

We noted one case of Guillain–Barre syndrome (within our IMID sample of 1556, representing 0.06%) and one case of Bell’s Palsy (within our IMID sample of 1556, representing 0.06%). When comparing to the general population, Frontera et al. reported that neurological adverse events, including Guillain–Barre syndrome and Bell’s Palsy, occurred in 0.03% of their study population [18]. Ogunjimi et al., in their meta-analysis of 70 papers, similarly reported a very low prevalence of Guillain–Barre syndrome, with a pooled prevalence of 8.1 per 1,000,000 vaccinations [19]. We also noted a case of a serious allergic reaction requiring an ED visit with facial edema. In the general population, anaphylaxis has been reported in 1 per 100,000 doses of vaccines administered [20]. We also had one participant with PsA who reported severe menstrual bleeding that required medical intervention. When compared to the general population, Wong et al. found that in their large observational cohort study, approximately 1% of 5,975,363 participants reported menstrual irregularities or vaginal bleeding [21]. We noted two cases of pericarditis following COVID-19 vaccination, with one likely being due to an SLE flare given the presence of other inflammatory symptoms. While the prevalence of isolated pericarditis in the general population post-vaccination remains unclear, the combined rate of myocarditis and pericarditis has been reported as 1.17 per 100,000 vaccine doses [20]. As mentioned, pericarditis may overlap with IMID flare symptoms.

Our findings from this study were presented and discussed with people with lived experiences of IMIDs. They pointed out that in 2021, many people with IMIDs had to reconcile the need for a vaccine that could potentially prevent infection (and possibly, hospital stays or even death) with the knowledge that there were very few early studies of adverse events after COVID-19 vaccination, and none in people with auto-immune diseases. People with IMIDs with jobs requiring them to physically report to work (e.g., in health care or other fields) would have had the extra pressure of needing to be vaccinated early in 2021 because of the increased risk of contracting COVID-19 despite some uncertainty regarding safety in individuals with an IMID. Since 2021, we have accumulated data on adverse events after COVID-19 vaccination in individuals with an IMID [12,13,14,15,16,17]. Of note, more recent variants (e.g., Omicron) have tended to cause somewhat less severe COVID-19 disease. A potential result may be that even in the face of PHAC/NACI recommendations to receive booster doses, some people with IMIDs may hesitate to get additional boosters beyond their third vaccine dose (which currently represents the ‘primary series’ in immunocompromised individuals).

Undoubtedly, a potential concern is that adverse events, including flares, may have been under-reported if individuals were comfortable self-managing their flares or if individuals were averse to presenting to the ED. This could be due to concerns regarding the wait time or exposure to COVID-19 infection, particularly in the 2021–2022 period when many individuals may not have had a COVID-19 infection yet and feared putting themselves at risk from sick COVID-19 patients in the ED. Another reason is that individuals with chronic conditions often prefer to present to their family physicians or rheumatologists who know them well rather than to the ED given their past health care interactions. Among the flares that occurred, some of these may have been due to individuals delaying their immunosuppressive medications for 1–2 weeks prior to getting a vaccine and/or not taking it for 1–2 weeks afterwards. We could not document this with our study design.

Naturally, there may be remaining concerns about long-term data on the effects (positive or negative) of multiple COVID-19 vaccinations in individuals with an IMID regarding disease control or comorbidity. People with IMIDs may have specific factors (e.g., past, concurrent, or family history of cardiac disease, demyelinating disorders, or thrombosis) that made them hesitant to get vaccinated in the past and/or may contribute to their hesitancy regarding future vaccination (e.g., in the setting of recommendations for booster vaccinations every 6–12 months). In addition, most North Americans have had one or more COVID-19 infections, and some people may feel that their antibody titres are high enough. Some people may also believe that since they have recovered from COVID-19 without sequelae, they would recover again. On the other hand, considering that people with IMIDs have an increased risk of hospitalization and intensive care unit stays for infections in general, some individuals with IMIDs may welcome regular boosters. This may also arise if they have a high risk of COVID-19 infection due to living with young children or exposure to grandchildren, or if they have family members who are also immunocompromised.

In terms of vaccine type and related adverse events, people with an IMID may very well have made choices on vaccine type due to evolving knowledge (across 2021) regarding specific adverse event associations in subgroup populations. Some of the adverse events include myocarditis in young men with mRNA vaccines [22] or thrombosis in people receiving early COVID-19 vaccine formulations [23] (which could be particularly of concern for individuals with an IMID with baseline risk factors for thrombosis, such as anti-phospholipid and related antibodies and/or past thrombosis).

One major strength of our study includes our large sample of participants with an IMID, with data gathered across eight Canadian sites, who are likely representative of the general population of individuals with IMID. In addition to involving people with lived experiences in the planning and execution of our research, our study is novel because we asked a group of people with lived experiences to help us interpret the results, which allowed their voices to be heard. This is extremely important, since our ultimate goal is to help optimize the uptake of COVID-19 vaccines through our results in an era where SARS-CoV-2 continues to circulate at the same time that social ‘vaccine fatigue’ or safety concerns may prevent people with IMIDs from continuing to get boosters.

Our study has some potential limitations. Firstly, although this study was launched only a few months after COVID-19 vaccinations were approved in Canada, many participants were only recruited after their second or subsequent vaccine doses. We asked the participants to still report information on their initial vaccines, including information on adverse events, even if they were enrolled after the second or subsequent doses. Because the participants had to provide information on those earlier doses, our results could be potentially subject to recall bias. Moreover, individuals who experienced an SAE following the initial vaccine doses may have been hesitant to proceed with subsequent doses. Additionally, we relied on participants’ written self-reports of their SAEs, which we confirmed by contacting each site’s study directors; however, we did not consult each subject’s health record to look for any unreported adverse events. This approach could introduce selection bias, as individuals with lower education or literacy skills might be less likely to participate or accurately report adverse events. Among those who reported an SAE, the levels of education were varied, with approximately 17% reporting a high school education, 25% reporting trade/apprenticeship training, 25% completing community college, 8.3% completing a bachelor’s degree, and 25% completing a graduate degree. These results closely resemble the educational background of the entire study population, where 76.8% of participants had at least a high school diploma and/or further education, with only 3.2% of participants reporting education less than a high school diploma. Given that a significant portion of our study population had completed high school at minimum, it is unlikely that literacy skills posed a substantial limitation in the reporting of adverse events. In addition, not all of our study sites collected information on individuals who refused to participate. At the Montreal center, 90% of the approached individuals consented to participate. Of those who declined, the reasons were primarily low energy/feeling unwell (about 22%) and other time commitments including travel or participating in another study (about 44%), and the remainder declined due to a lack of interest. Unfortunately, our ethics committee did not permit us to specifically collect or analyze data on individuals who refused to participate. Thus, we are unable to fully exclude the possibility of selection bias. This limitation is also present in other similar studies on the topic of COVID-19 vaccination in individuals with an IMID [8,24,25]. We presented the results of this study to patients’ partners who agreed that our sample is representative of their patient group. Furthermore, in this study, we only explored events which are temporally associated with COVID-19 vaccination. We did not explore any potential long-term SAEs due to the challenges in distinguishing these events from complications of underlying medical conditions. In addition, although we compared our findings to what has already been reported in the general population in the literature, our study did not specifically collect or analyze data on a control group without IMIDs. Lastly, in our sample, we analyzed data from participants who received monovalent vaccines which had no specificity for Omicron variants.

In conclusion, within the 31 days after SARS-CoV-2 vaccination in individuals with an IMID, there were relatively few SAEs and no deaths.

## Figures and Tables

**Table 1:** Baseline participant characteristics/demographics.

Characteristic	Total N = 1556 (%)
**Age, N (%)**	
≤60 years	1014 (65.2)
60+ years	533 (34.3)
Not specified	9 (0.6)
Mean age (SD)	52.5 (15.7)
**Sex, N (%)**	
Female	978 (62.9)
Male	569 (36.6)
Not specified	9 (0.6)
**White race/ethnicity, N (%)**	1343 (86.3)
**Mean disease duration (SD) (years)**	17.1 (13.4)
**Current smoker, N (%)**	78 (5.1)
**Disease, N (%)**	
Inflammatory bowel disease	763 (49.0)
Rheumatoid arthritis	426 (27.4)
Psoriasis/psoriatic arthritis	223 (14.3)
Axial spondylarthritis	82 (5.3)
Systemic lupus erythematosus	62 (4.0)
**Current prednisone use, N (%)**	308 (19.8)
**Current biologic drug use, N (%)**	
Current anti-TNF	551 (35.4)
Current ustekinumab	170 (10.9)
Current vedolizumab	100 (6.4)
Current other biologic drug	46 (3.0)
Current abatacept	39 (2.5)
Current rituximab	17 (1.1)
No biologic drug	496 (31.9)
**Non-biologic drug use, N (%)**	
Current methotrexate	439 (28.2)
Current hydroxychloroquine	234 (15.0)
Current azathioprine	90 (5.8)
Current sulfasalazine	89 (5.7)
Current jak-inhibitor	86 (5.5)
Current leflunomide	56 (3.6)
Current other drug	8 (0.5)
**Number vaccines, N (%)**	
One dose	43 (2.8)
Two doses	223 (14.3)
Three doses	675 (43.4)
Four doses	531 (34.1)
Five doses	84 (5.4)
**Vaccine Type, N (%)**	
BNT-162b2 monovalent	1018 (65.4)
mRNA1273 monovalent	223 (14.3)
Mixed BNT-162b2/mRNA1273	262 (16.8)
Non-mRNA vaccine ^a^	53 (3.4)

^a^ These patients are those who received a single dose of either ChAdOx1 nCoV-19 or Ad26.COV2.S or those who received two doses of ChAdOx1 nCoV-19.

**Table 2:** Severe, self-reported vaccine-related adverse events and severe disease flare within 31 days of each COVID-19 vaccine dose.

	Dose 1 N = 1556	Dose 2 N = 1506	Dose 3 N = 1278	Dose 4 N = 597	Dose 5 N = 50
**Requiring emergency department, ED visit only (no admissions)**					
Neurologic events ^a^	1	0	0	0	0
Thrombosis	0	0	0	0	0
Pericarditis	0	1	0	0	0
Disease flare ^b^	0	0	1	0	0
Other ^c^	1	1	1	0	0
Total	2	2	2	0	0
**Requiring Hospitalization (+/−ED)**					
Neurologic events ^a^	1	0	0	0	0
Thrombosis	0	0	0	0	0
Disease flare	0	0	0	0	0
Other ^d^	0	1	3	1	0
Total	1	1	3	1	0

^a^ Neurologic events include the following: Bell’s Palsy (1), Guillain–Barre syndrome (1). ^b^ Disease flare occurred in 1 SLE patient post dose 3. ^c^ Other reasons for ED visits include the following: severe allergic reaction (1), labyrinthitis (1), and severe menstrual bleeding (1). ^d^ Other reasons for hospitalization include the following: diverticulosis (1), idiopathic thrombocytopenia purpura (1), transient multifactorial renal failure (1), atrial fibrillation (1), and migraine with aura (1).

**Table 3:** Baseline immune-mediated inflammatory diseases, medications, and outcomes of each severe adverse event experienced by participants.

Participant	Severe Adverse Event(s)	Emergency Department (ED)/Hospitalization	Severe Adverse Event Outcome	Baseline Disease	Baseline Medications and Medications Used Prior Study Entry *
1	Bell’s Palsy	ED	Fully recovered	Axial spondylarthritis	Golimumab
2	Severe allergic reaction	ED	Fully recovered	Systemic lupus erythematosus	Prednisone 1 mg die
3	1. Labyrinthitis 2. Pericarditis-possible flare	ED	Fully recovered	Systemic lupus erythematosus	Prednisone 50 mg die
4	Severe menstrual bleeding	ED	Fully recovered	Psoriatic arthritis	Ustekinumab
5	Pericarditis	ED	Fully recovered	Rheumatoid arthritis	Methotrexate; past use of hydroxychloroquine
6	Guillain–Barre syndrome	Hospitalization	Permanent disability/incapacity	Rheumatoid arthritis	Prednisone 2.5 mg die, methotrexate, adalimumab
7	Idiopathic thrombocytopenic purpura	Hospitalization	Unknown	Inflammatory bowel disease	Methotrexate, infliximab
8	Atrial fibrillation	Hospitalization	Fully recovered	Rheumatoid arthritis	Methotrexate, hydroxychloroquine, adalimumab; past use of prednisone
9	Transient multifactorial renal failure	Hospitalization	Not yet fully recovered	Psoriatic arthritis	Adalimumab
10	Migraine with aura	Hospitalization	Fully recovered	Rheumatoid arthritis	Hydroxychloroquine, etanercept; past use of prednisone
11	Diverticulosis	Hospitalization	Fully recovered	Systemic lupus erythematosus	None indicated
12	Shingles	Hospitalization	Fully recovered	Rheumatoid arthritis	Prednisone 7.5 mg die, methotrexate; past use of hydroxychloroquine
13	Epiploic appendagitis	Hospitalization	Fully recovered	Rheumatoid arthritis	Prednisone 7.5 mg die, abatacept; past use of methotrexate, hydroxychloroquine, leflunomide, sulfasalazine

* The medication list only included medications participants took for their baseline immune-mediated inflammatory diseases and is not an exhaustive list of all of their medications.

## Data Availability

The data are contained within the article. The original contributions presented in this study are included in this article, and further inquiries can be directed to the corresponding author.

## References

[1] Polack F.P., Thomas S.J., Kitchin N., Absalon J., Gurtman A., Lockhart S., Perez J.L., Pérez Marc G., Moreira E.D., Zerbini C. (2020). Safety and Efficacy of the BNT162b2 mRNA Covid-19 Vaccine. N. Engl. J. Med..

[2] Baden L.R., El Sahly H.M., Essink B., Kotloff K., Frey S., Novak R., Diemert D., Spector S.A., Rouphael N., Creech C.B. (2021). Efficacy and Safety of the mRNA-1273 SARS-CoV-2 Vaccine. N. Engl. J. Med..

[3] Krammer F. (2020). SARS-CoV-2 vaccines in development. Nature.

[4] Eder L., Widdifield J., Rosen C.F., Cook R., Lee K., Alhusayen R., Paterson M.J., Cheng S.Y., Jabbari S., Campbell W. (2019). Trends in the Prevalence and Incidence of Psoriasis and Psoriatic Arthritis in Ontario, Canada: A Population-Based Study. Arthritis. Care. Res..

[5] Ng S.C., Shi H.Y., Hamidi N., Underwood F.E., Tang W., Benchimol E.I., Panaccione R., Ghosh S., Wu J.C.Y., Chan F.K.L. (2017). Worldwide incidence and prevalence of inflammatory bowel disease in the 21st century: A systematic review of population-based studies. Lancet.

[6] Widdifield J., Paterson J.M., Bernatsky S., Tu K., Tomlinson G., Kuriya B., Thorne J.C., Bombardier C. (2014). The epidemiology of rheumatoid arthritis in Ontario, Canada. Arthritis. Rheumatol..

[7] MacKenna B., AKennedy N., Mehrkar A., Rowan A., Galloway J., Matthewman J., Mansfield K.E., Bechman K., Yates M., Brown J. (2022). Risk of severe COVID-19 outcomes associated with immune-mediated inflammatory diseases and immune-modifying therapies: A nationwide cohort study in the OpenSAFELY platform. Lancet Rheumatol..

[8] Markovinovic A., Herauf M., Quan J., Hracs L., Windsor J.W., Sharifi N., Coward S., Caplan L., Gorospe J., Ma C. (2023). Adverse Events and Serological Responses After SARS-CoV-2 Vaccination in Individuals with Inflammatory Bowel Disease. Am. J. Gastroenterol..

[9] Vaccine Vigilance Working Group & PHAC (2023). Reporting Adverse Events Following Immunization (AEFI) in Canada: User Guide to Completion and Submission of the AEFI Reports. Government of Canada. https://www.canada.ca/en/public-health/services/immunization/reporting-adverse-events-following-immunization/user-guide-completion-submission-aefi-reports.html.

[10] Yasmin F., Najeeb H., Naeem U., Moeed A., Atif A.R., Asghar M.S., Nimri N., Saleem M., Bandyopadhyay D., Krittanawong C. (2023). Adverse events following COVID-19 mRNA vaccines: A systematic review of cardiovascular complication, thrombosis, and thrombocytopenia. Immun. Inflamm. Dis..

[11] Fraiman J., Erviti J., Jones M., Greenland S., Whelan P., Kaplan R.M., Doshi P. (2022). Serious adverse events of special interest following mRNA COVID-19 vaccination in randomized trials in adults. Vaccine.

[12] Bartels L.E., Ammitzbøll C., Andersen J.B., Vils S.R., Mistegaard C.E., Johannsen A.D., Hermansen M.-L.F., Thomsen M.K., Erikstrup C., Hauge E.-M. (2021). Local and systemic reactogenicity of COVID-19 vaccine BNT162b2 in patients with systemic lupus erythematosus and rheumatoid arthritis. Rheumatol. Int..

[13] Naveen R., Parodis I., Joshi M., Sen P., Lindblom J., Agarwal V., Lilleker J.B., Tan A.L., Nune A., Shinjo S.K. (2023). COVID-19 vaccination in autoimmune diseases (COVAD) study: Vaccine safety and tolerance in rheumatoid arthritis. Rheumatology.

[14] Botwin G.J., Li D., Figueiredo J., Cheng S., Braun J., McGovern D.P.B., Melmed G.Y. (2021). Adverse Events After SARS-CoV-2 mRNA Vaccination Among Patients With Inflammatory Bowel Disease. Am. J. Gastroenterol..

[15] Pellegrino R., Pellino G., Selvaggi L., Selvaggi F., Federico A., Romano M., Gravina A.G. (2022). BNT162b2 mRNA COVID-19 vaccine is safe in a setting of patients on biologic therapy with inflammatory bowel diseases: A monocentric real-life study. Expert. Rev. Clin. Pharmacol..

[16] Cruz V.A., Guimarães C., Rêgo J., Machado K.L.L.L., Miyamoto S.T., Burian A.P.N., Dias L.H., Pretti F.Z., Batista D.C.F.A., Mill J.G. (2024). Safety of CoronaVac and ChAdOx1 vaccines against SARS-CoV-2 in patients with rheumatoid arthritis: Data from the Brazilian multicentric study SAFER. J. Rheumatol..

[17] Isnardi C.A., Schneeberger E.E., Kreimer J.L., Luna P.C., Echeverría C., Roberts K., de la Vega M.C., Virasoro B.M., Landi M., Quintana R. (2022). An Argentinean cohort of patients with rheumatic and immune-mediated diseases vaccinated for SARS-CoV-2: The SAR-CoVAC Registry-protocol and preliminary data. Clin. Rheumatol..

[18] Frontera J.A., Tamborska A.A., Doheim M.F., Garcia-Azorin D., Gezegen H., Guekht A., Khan Y.K.A., Santacatterina M., Sejvar J., Thakur K.T. (2022). contributors from the Global COVID-19 Neuro Research Coalition. Neurological events reported after COVID-19 vaccines: An analysis of VAERS. Ann. Neurol..

[19] Ogunjimi O.B., Tsalamandris G., Paladini A., Varrassi G., Zis P. (2023). Guillain-Barré syndrome induced by vaccination against COVID-19: A systematic review and meta-analysis. Cureus.

[20] Government of Canada (2024). Reported Side Effects Following COVID-19 Vaccination in Canada. https://health-infobase.canada.ca/covid-19/vaccine-safety/.

[21] Wong K.K., Heilig C.M., Hause A., Myers T.R., Olson C.K., Gee J., Marquez P., Strid P., Shay D.K. (2022). Menstrual irregularities and vaginal bleeding after COVID-19 vaccination reported to v-safe active surveillance, USA in December, 2020-January, 2022: An observational cohort study. Lancet. Digit. Health.

[22] Knudsen B., Prasad V. (2023). COVID-19 vaccine induced myocarditis in young males: A systematic review. Eur. J. Clin. Investig..

[23] D’almeida S., Markovic S., Hermann P., Bracht H., Peifer J., Ettrich T.J., Imhof A., Zhou S., Weiss M., Viardot A. (2023). Thromboembolism after Astra Zeneca COVID-19 vaccine: Not always PF4- antibody mediated. Hum. Vaccines. Immunother..

[24] Cheung M.W., Dayam R.M., Shapiro J.R., Law J.C., Chao G.Y.C., Pereira D., Goetgebuer R.L., Croitoru D., Stempak J.M., Acheampong L. (2023). Third and Fourth Vaccine Doses Broaden and Prolong Immunity to SARS-CoV-2 in Adult Patients with Immune-Mediated Inflammatory Diseases. J. Immunol..

[25] Benoit J.M., Breznik J.A., Ang J.C., Bhakta H., Huynh A., Cowbrough B., Baker B., Heessels L., Lodhi S., Yan E. (2023). Immunomodulatory drugs have divergent effects on humoral and cellular immune responses to SARS-CoV-2 vaccination in people living with rheumatoid arthritis. Sci. Rep..

